# Identification of viruses infecting six plum cultivars in Korea by RNA-sequencing

**DOI:** 10.7717/peerj.9588

**Published:** 2020-07-29

**Authors:** Yeonhwa Jo, Hoseong Choi, Sen Lian, Jin Kyong Cho, Hyosub Chu, Won Kyong Cho

**Affiliations:** 1 Research Institute of Agriculture and Life Sciences, College of Agriculture and Life Sciences, Seoul National University, Seoul, Republic of Korea; 2 Department of Agricultural Biotechnology, College of Agriculture and Life Sciences, Seoul National University, Seoul, Republic of Korea; 3 College of Crop Protection and Agronomy, Qingdao Agricultural University, Qingdao, China; 4 Won Kyong Farm, Hoengseong, Republic of Korea; 5 Core Protein Resources Center, Daegu Gyeongbuk Institute of Science and Technology, Daegu, Republic of Korea

**Keywords:** Plum, RNA-seq, Viroid, Virus, Korea

## Abstract

**Background:**

Plums are a kind of stone fruit, a category that includes peaches, cherries, apricots, and almonds. In Korea, Japanese plum trees are usually cultivated as they best suit the climate. To date, there have been few studies in Korea on viruses infecting plum trees compared to those infecting peach trees.

**Methods:**

To identify viruses and viroids infecting plum trees, we collected leaf samples from six different plum cultivars and subjected them to RNA-sequencing (RNA-seq). Six different plum transcriptomes were de novo assembled using the Trinity assembler followed by BLAST searching against a viral reference database.

**Results:**

We identified hop stunt viroid (HSVd) and six viruses, including apple chlorotic leaf spot virus (ACLSV), little cherry virus-1 (LChV-1), peach virus D (PeVD), peach leaf pitting-associated virus (PLPaV), plum bark necrosis stem pitting-associated virus (PBNSPaV), and prunus necrotic ringspot virus (PNRSV), from six plum cultivars by RNA-seq. RT-PCR confirmed the infection of HSVd and three viruses—ACLSV, PBNSPaV, and PNRSV—in plum trees. However, RT-PCR demonstrated that plum trees in this study were not infected by LChV-1, PeVD, or PLPaV. It is likely that the three viruses LChV-1, PeVD, and PLPaV as identified by RNA-seq were contaminants from other peach libraries caused by index misassignment, which suggests that careful confirmation by other methods should be carried out in next-generation sequencing (NGS)-based virus identification. Taken together, we identified a viroid and three viruses infecting plum trees in Korea.

## Introduction

Plums are a kind of stone fruit, a category that includes peaches, cherries, apricots, and almonds (Yılmaz et al., 2009; Zhebentyayeva et al., 2019). The plum is usually consumed as fresh fruit; however, it can also be canned, dried, or used to make jam and wine. In general, two different plum species, the hexaploid European plum (*Prunus domestica*) (Zhebentyayeva et al., 2019) and the diploid Japanese plum (*Prunus salicina* and hybrids), are widely grown (Mnejja et al., 2004).

Most well-known plum cultivars are clonally propagated by grafting, which can cause them to be co-infected by several viruses and viroids. Of the known viral agents infecting plum trees, *Plum pox virus* (PPV) in the genus *Potyvirus* causing Sharka disease is the most destructive (García et al., 2014). In addition, *Plum bark necrosis stem pitting-associated virus* (PBNSPaV) in the genus *Ampelovirus* (Amenduni et al., 2005) and *Hop stunt viroid* (HSVd) in the genus *Hostuviroid* are known to infect plum trees (Jo et al., 2017b; Sano et al., 1989).

Currently, next-generation sequencing (NGS)-based approaches are widely used in the identification of viruses infecting plants (Barba, Czosnek & Hadidi, 2014; Hadidi et al., 2016; Ho & Tzanetakis, 2014; Massart et al., 2014; Roossinck, Martin & Roumagnac, 2015). NGS techniques can identify not only known viruses and viroids but also novel viruses; numerous novel viruses are now being identified using NGS techniques. In addition, studies associated with the viral genome, a complex of virus populations, viral mutation, and evolution have been conducted using NGS and bioinformatics (Jo et al., 2015, 2017a, 2018b).

For NGS, plant leaf samples with obvious symptoms are harvested and subjected to nucleic acid extraction and libraries are prepared from extracted nucleic acids. The most frequently used methods for library preparation are double-stranded (ds) RNA library, mRNA library, ribosome-depleted RNA library, and small RNA library. Two recent studies compared different viral sequence enrichment methods, suggesting that the yield of viral sequences by NGS was dependent on viral genome organizations and the number of viral reads in the data (Ma et al., 2019; Pecman et al., 2017).

Japanese plum trees are usually cultivated in Korea as they best suit the climate. Most plum cultivars in Korea have been derived from Japan or other countries. Although several native plum species called *goya* or *oyat* are grown in Korea, their fruits are relatively smaller with diverse fruit colors. It seems that the native plum species in Korea might be propagated by seedlings.

To date, there have been few studies in Korea on viruses infecting plum trees compared to those infecting peach trees. Only two viruses, *Prunus necrotic ringspot virus* (PNRSV) in the genus *Ilarvirus* and *Apple chlorotic leaf spot virus* (ACLSV) in the genus *Trichovirus*, and HSVd infecting Japanese plum trees have been reported in Korea (Jo et al., 2016). In this study, we identified viruses and viroids infecting six different plum cultivars representing three *Prunus* species by RNA-sequencing (RNA-seq).

## Materials and Methods

### Plant materials and total RNA extraction

Leaf samples from five plum cultivars—Akihime, Formosa, Hollywood, Gadam, and Yeonsik—grown in an orchard in Hoengseong, Korea were harvested in May 2013 and leaf samples from a European plum (unknown cultivar) were harvested in September 2012. The plum trees had been properly sprayed with agricultural pesticides such as insecticides and fungicides. We collected five leaves from each plant representing a cultivar. Leaf samples were combined and subjected to total RNA extraction using Fruit-mate for RNA Purification (Takara, Shiga, Japan) and the RNeasy Plant Mini Kit for RNA extraction (Qiagen, Hilden, Germany), following the manufacturers’ instructions. The quality and quantity of extracted RNA were measured by gel electrophoresis and NanoDrop Spectrophotometers (Thermo Fisher Scientific, Waltham, MA, USA).

### Preparation of libraries for RNA-seq

In this study, we generated six mRNA libraries derived from the six different plum trees. We extracted mRNAs poly-A tail using Oligo d(T)25 Magnetic Beads (NEB, Ipswich, MA, USA) according to the manufacturer’s instructions. The mRNA libraries were prepared using the TruSeq RNA Library Preparation Kit v2 (Illumina, CA, USA) following the manufacturer’s instructions. The quality control of the generated libraries was carried out using a 2100 Bioanalyzer (Agilent, Santa Clara, USA). The average insert size was 350 bp. The six plum libraries were paired-end (2 × 100 bp) sequenced by Macrogen Co. (Seoul, South Korea) using the HiSeq 2000 platform. In order to reduce the cost for NGS, six plum libraries in this study and six peach libraries in the previous studies (Jo et al., 2019, 2018b) were sequenced in a single lane. All raw sequences were deposited in the SRA database in NCBI with respective accession numbers under BioProject PRJNA295439. Using the PRINSEQ program (Schmieder & Edwards, 2011), we obtained clean reads from each library that were further subjected to de novo transcriptome assembly.

### De novo transcriptome assembly

For de novo transcriptome assembly, we used Trinity (version 2.0.2, released January 22, 2015) (Haas et al., 2013) with default parameters. The K-mer size for Trinity was 21 and the minimum contig length for Trinity is 200 nucleotides (nt). Two paired-end sequenced FASTQ files for each individual library were used for de novo transcriptome assembly with default parameters. A workstation with two six-core CPUs and 256 GB RAM operated using the Ubuntu 12.04.5 LTS operation system was used for bioinformatics analyses. Finally, we obtained six transcriptomes from the six libraries.

### Identification of viruses and viroids by BLAST search

To identify viruses and viroids in the assembled plum transcriptomes, we carried out the MEGABLAST algorithm with a cutoff E-value of 1e^−10^. We only extracted the genome sequences of plant viruses and viroids from the NCBI’s reference viral genome database (http://www.ncbi.nlm.nih.gov/genome/viruses/) and we established an in-house plant viral genome database. The assembled contigs from each plum transcriptome were blasted against a database composed of plant viruses and viroids. In addition, we conducted BLASTX with a cutoff E-value of 1e^–3^ using the assembled transcriptome against the viral protein database. The identified contigs associated with viral sequences were used for a BLASTX search against an NCBI non-redundant proteins (NR) database to remove sequences derived from the plum hosts. Finally, we obtained clean virus-associated contigs from each plum transcriptome.

### RT-PCR and cloning of PCR products

To confirm the presence of identified viruses in the six plum samples, RT-PCR was carried out using virus-specific primers (Table 1). We performed one-step RT-PCR using the DiaStar OneStep RT-PCR Kit (SolGent, Daejeon, Korea). In brief, one µg of total RNA and 10 pmoles of each reverse primer was combined to a total volume of 30 µl. RT-PCR was conducted using a C1000™ thermal cycler (BIO-RAD, Hercules, USA) as follows: 50 °C for 30 min and 95 °C for 15 min; followed by 35 cycles of denaturation at 95 °C for 20 s, annealing at 50–55 °C for 40 s, and extension at 72 °C for 30 s; there followed a final extension at 72 °C for 5 min. We examined the size of the amplified RT-PCR product in 1% TAE agarose gel by staining with EtBr for UV visualization. The individual amplified PCR product was cloned into pGEM-T-Easy Vector (Promega, Wisconsin, USA) followed by Sanger sequencing. The sequencing results were used for the BLASTN search against NCBI’s NT database.

**Table 1:** Information of primers used for RT-PCR.

Name of virus	Primer name	Primer sequences (5′ to 3′)	Size of amplicon
ACLSV	ACLSV-cp-F1	ATGGCAGCAGTTCTGAATTTGC	582 bp
	ACLSV-cp-R1	CTAAATGCAAAGATCAGTCGACACAGA	
PNRSV	PNRSVCP-F	AACTGCAGATGGTTTGCCGAATTTGCAA	675 bp
	PNRSVCP-R	GCTCTAGACTAGATCTCAAGCAGGTC	
PBNSPaV	PBNSPaV_F1	TACCGAAGAGGGTTTGGATG	400 bp
	PBNSPaV_R1	AGTCGCACCACCAGTCTTCT	
18S rRNA	18sPruF1	CGTCACACGCCGTTGCCCCC	199 bp
	18sPruR1	GAGCCGAGCATTTTTTCGAGCCC	

**Note:**

Primers for PNRSV, PBNSPaV, and 18S rRNA were derived from previous studies (Al Rwahnih et al. 2007; Jarošová & Kundu 2010; Kulshrestha et al. 2013).

### Sequence alignment and phylogenetic analyses

Full-length amino acid sequences for the coat protein (CP) and movement protein (MP) of ACLSV and the CP and MP of PNRSV were subjected to BLASTP search against the NR database. Homologous viral protein sequences were retrieved and subjected to sequence alignment using MAFFT ver. 7.310 (March 17, 2017) with the G-INS-I (accurate) strategy (Katoh & Standley, 2013). Aligned sequences were trimmed by the trimAL program with the automated method (http://trimal.cgenomics.org/). The best-fitting substitution model for each aligned protein sequence was selected using IQ-TREE (Nguyen et al., 2014). The phylogenetic tree was inferred using the IQ-TREE program with the maximum likelihood method, selected substitution model, and ultrafast bootstrap according to the manufacturers’ instructions (Hoang et al., 2017). FigTree was used to visualize the phylogenetic tree (http://tree.bio.ed.ac.uk/software/figtree/).

## Results

### Sample collection and library preparation

**Figure 1:**
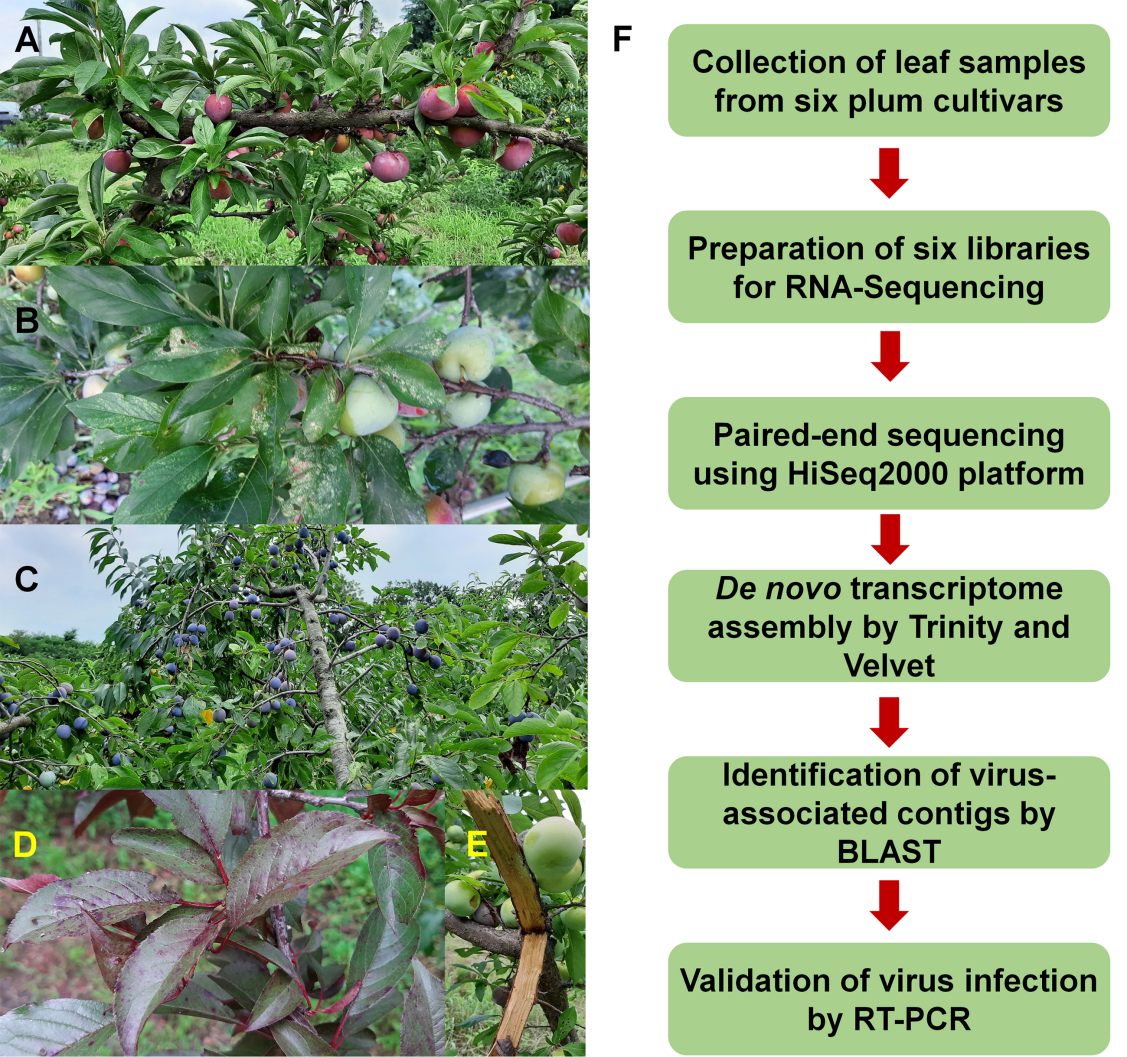
Images of plum trees and experimental scheme. (A) Healthy tree of plum cultivar Formosa. (B) Leaves and fruits of plum cultivar Gadam. (C) European plum tree. (D) Red colored leaves of plum cultivar Hollywood showing viral disease symptoms. (E) Bark of plum cultivar Akihime without any disease symptoms. (F) Experimental scheme for identification of viruses and viroids infecting plum.

To identify viruses infecting different plum cultivars, we selected six representative cultivars: Akihime (*P. salicina*) (AH), Gadam (*P. salicina*) (PG), Yeonsik (*P. salicina*) (PY), Hollywood (*P. cerasifera*) (HW), an unknown European plum (*P. domestica*) (EP), and Formosa (*P. salicina*) (FM) (Figs. 1A–1E). We collected leaf samples from individual cultivars. Most plum cultivars did not show any visible disease symptoms; however, PG and HW showed viral disease symptoms in the leaves (Figs. 1B and 1D). We have prepared six libraries representing the six cultivars. A total of 12 libraries including the six plum libraries in this study and the six peach libraries in the previous studies (Jo et al., 2019, 2018b) were paired-end sequenced in a single lane using the HiSeq2000 platform to minimize the cost for NGS.

### Identification of viruses and viroids from plum transcriptomes

**Figure 2:**
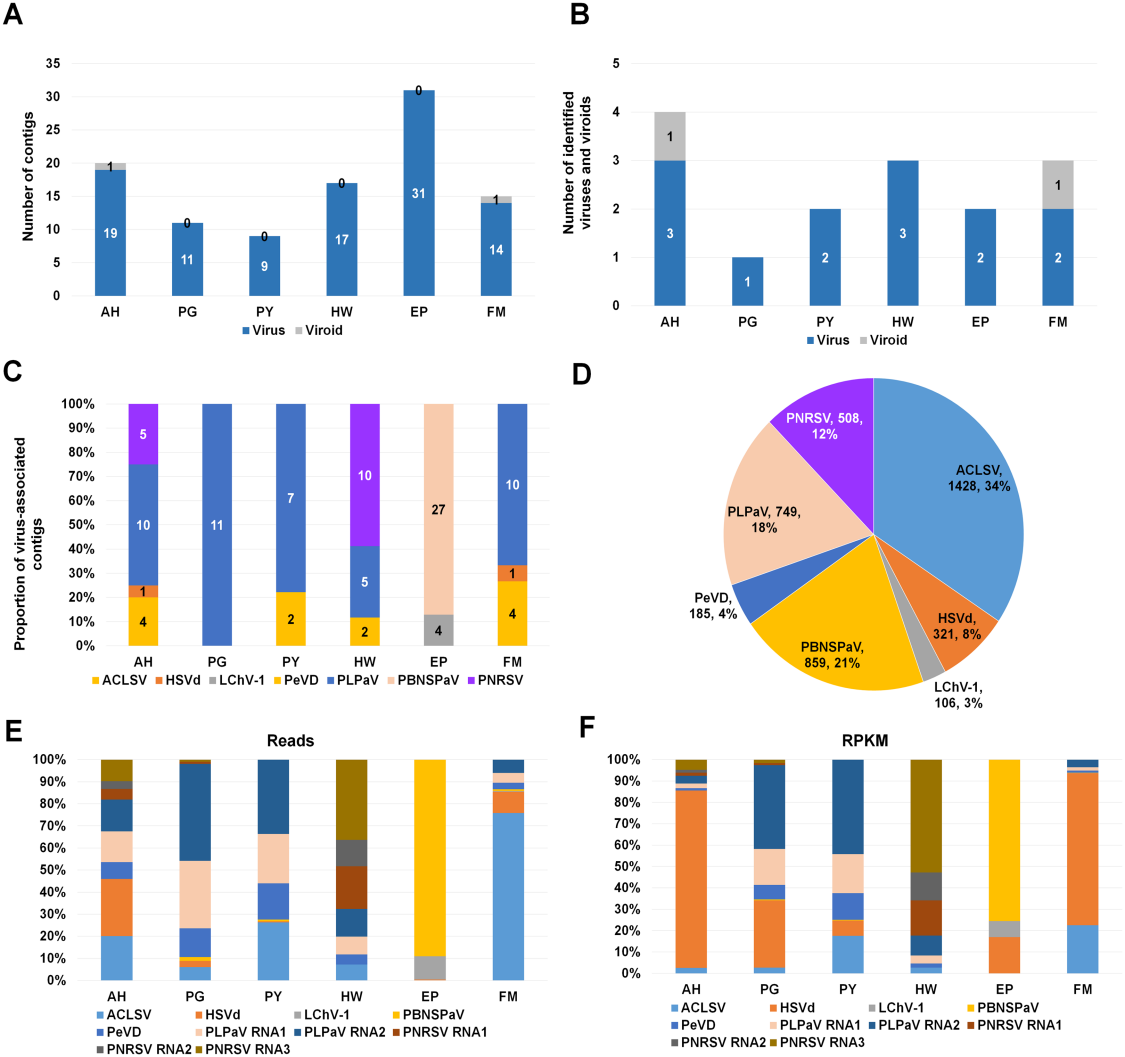
Identification of viruses and viroids infecting plum cultivars by RNA-seq. (A) Numbers of virus-associated contigs (Green color) and viroid-associated contigs (Red color) from six plum transcriptomes. AH, PG, PY, HW, EP, and FM indicate abbreviated names of different plum cultivars. (B) Numbers of identified viruses (green color) and viroids (red color) from six different plum cultivars. (C) Proportion of identified viruses and a viroid in each plum transcriptome based on the number of identified virus-associated contigs. (D) Proportion of all viruses and a viroid identified from six plum transcriptomes based on viral reads. Virus-associated reads from each plum transcriptome were combined and classified according to the identified virus and viroid. (E) Proportion of viral populations based on viral RNAs in six plum cultivars. (F) Proportion of viral populations based on coverage in six plum cultivars.

To identify viruses and viroids infecting plum in detail, we used the Trinity assembler. The clean reads obtained from each library were further subjected to de novo transcriptome assembly (Table S1). The number of contigs associated with viruses or a viroid was varied for each library (Table S2). For example, the number of virus-associated contigs assembled by Trinity ranged from 9 (PY) to 31 (EP) (Fig. 2A). Only a single viroid-associated contig was identified from AH and FM (Fig. 2A).

Next, we examined the number of identified viruses and viroids in each plum cultivar (Fig. 2B). We identified a viroid from AH and FM, three different viruses from AH and HW, and two different viruses from PY, EP, and FM. There was a single virus infection in PG. Based on the number of virus- or viroid-associated contigs, we examined the proportion of the individual viruses and viroids in each plum transcriptome (Fig. 2C). In the four transcriptomes AH, PG, PY, and FM, the contigs associated with peach leaf pitting-associated virus (PLPaV) were dominant. PNRSV was a dominant virus In HW and PBNSPaV was a dominant virus in EP.

After combining all virus- and viroid-associated reads, we had identified six viruses and a viroid (HSVd) (Fig. 2D). Among these six viruses, ACLSV (34%) was the dominant virus followed by plum bark necrosis and stem pitting-associated virus (PBNSPaV) (21%), PLPaV (18%), PNRSV (12%), peach virus D (PeVD) (4%), and little cherry virus-1 (LChV-1) (3%).

### Viral populations in each plum transcriptome

As the plum trees were also co-infected by diverse viruses and a viroid, we decided to examine viral populations based on viral reads and reads per kilobase million (RPKM) values. To calculate viral reads, raw sequence reads were mapped on the individual reference viral genome using the Burrows–Wheeler Aligner (BWA) program. The mapped SAM file was subjected to the BBMap program to calculate viral reads. Based on viral RNAs, the dominant virus and viroid in AH were PLPaV and HSVd, respectively (Fig. 2E). PLPaV was also the dominant virus in PG and PY. In HW, the PNRSV-associated reads (392 reads) were abundantly present. PBNSPaV and ACLSV were the dominant viruses in EP and FM, respectively.

It is important to calculate viral abundance according to the viral genome size. For that, we calculated the RPKM values for each virus and viroid using BBMap program. Based on the RPKM values, HSVd (96%) was overwhelmingly the major viral pathogen infecting AH and FM (Fig. 2F). In PG and PY, PLPaV was again the dominant virus. In EP, PBNSPaV was dominant followed by HSVd. The proportion of HSVd was increased in five plum transcriptomes based on RPKM values.

### Confirmation of identified viruses by RT-PCR

**Figure 3:**
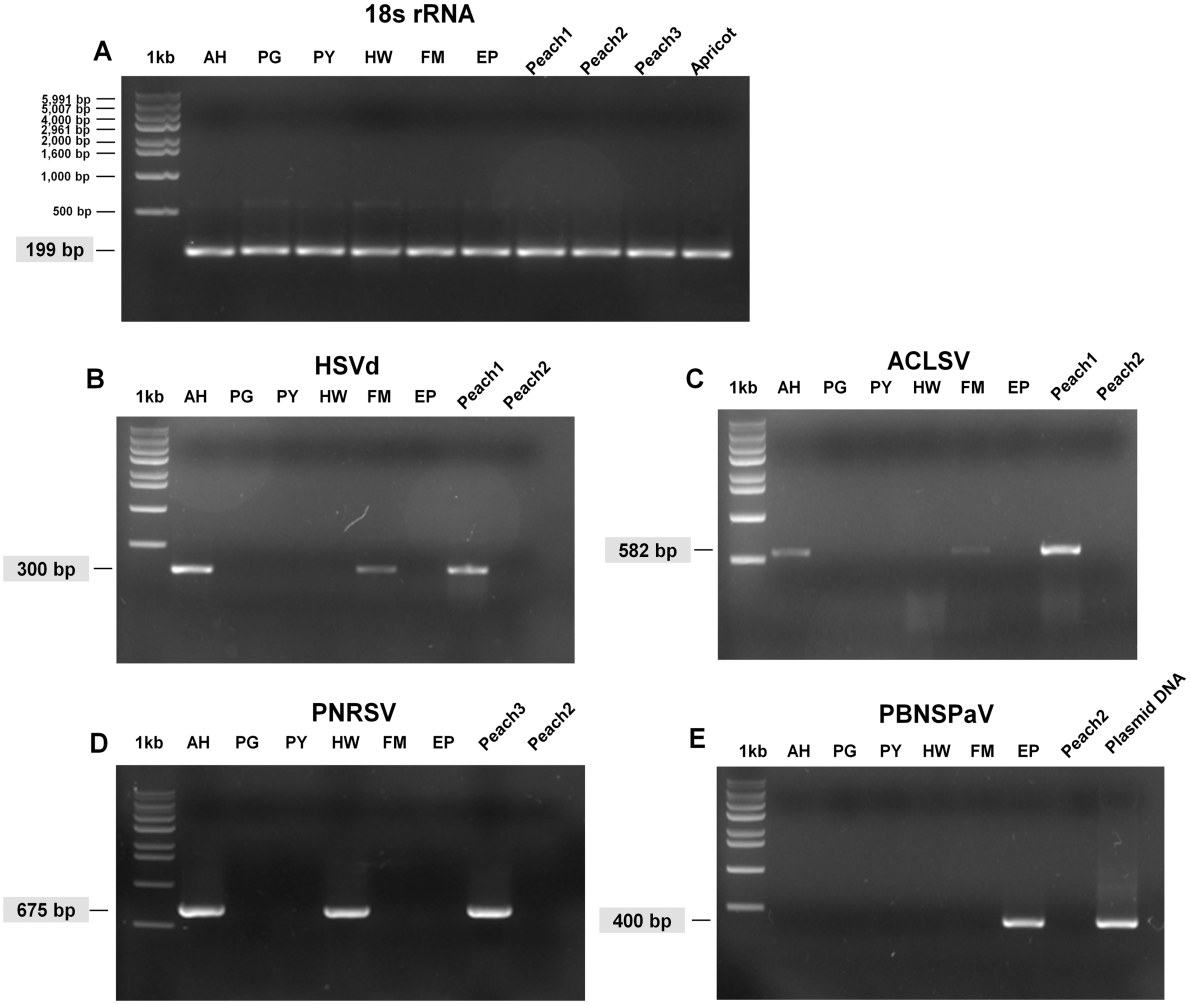
Full-length gels display RT-PCR results with specific primers for HSVd, ACLSV, PNRSV, and PBNSPaV. (A) RT-PCR results with the 18S rRNA specific primers showed the quality of extracted RNAs. The sizes of one kb DNA ladder were indicated (Bioneer, Daejeon, Korea). RT-PCR results with HSVd-specific primers (B), ACLSV-specific primers (C), PNRSV-specific primers (D), and PBNSPaV-specific primers (E). Peach1 was infected by HSVd and ACLSV while Peach2 used as a negative control was not infected by any virus or viroid. PBNSPaV plasmid DNA was used as a positive control. All PCR products were cloned and sequenced. The size of amplified PCR product was indicated.

We carried out RT-PCR to confirm the presence of viruses identified by RNA-seq. We have previously confirmed that HSVd infects plum trees by RT-PCR (Jo et al., 2017b). For RT-PCR, we designed primers for ACLSV while using previously known primers for PNRSV, PBNSPaV, and 18S rRNA (Table 1). We used primers for 18S rRNA to measure the quality of extracted total RNAs. We successfully amplified the PCR products for 18S rRNA from all samples (Fig. 3A). By RT-PCR, we confirmed the infection of HSVd in AH and FM (Fig. 3B) and the infection of ACLSV in AH and EP (Fig. 3C). In addition, infection of PNRSV in AH and infection of PBNSPaV in EP were confirmed by RT-PCR. However, we did not obtain any amplicon in the six plum trees for LChV-1, PeVD, or PLPaV by RT-PCR, which was repeated several times with positive and negative controls. We carefully supposed that the sequence reads associated with LChV-1, PeVD, and PLPaV were derived from other peach libraries, which were sequenced together with the plum libraries.

### Phylogenetic relationships of ACLSV and PNRSV

**Figure 4:**
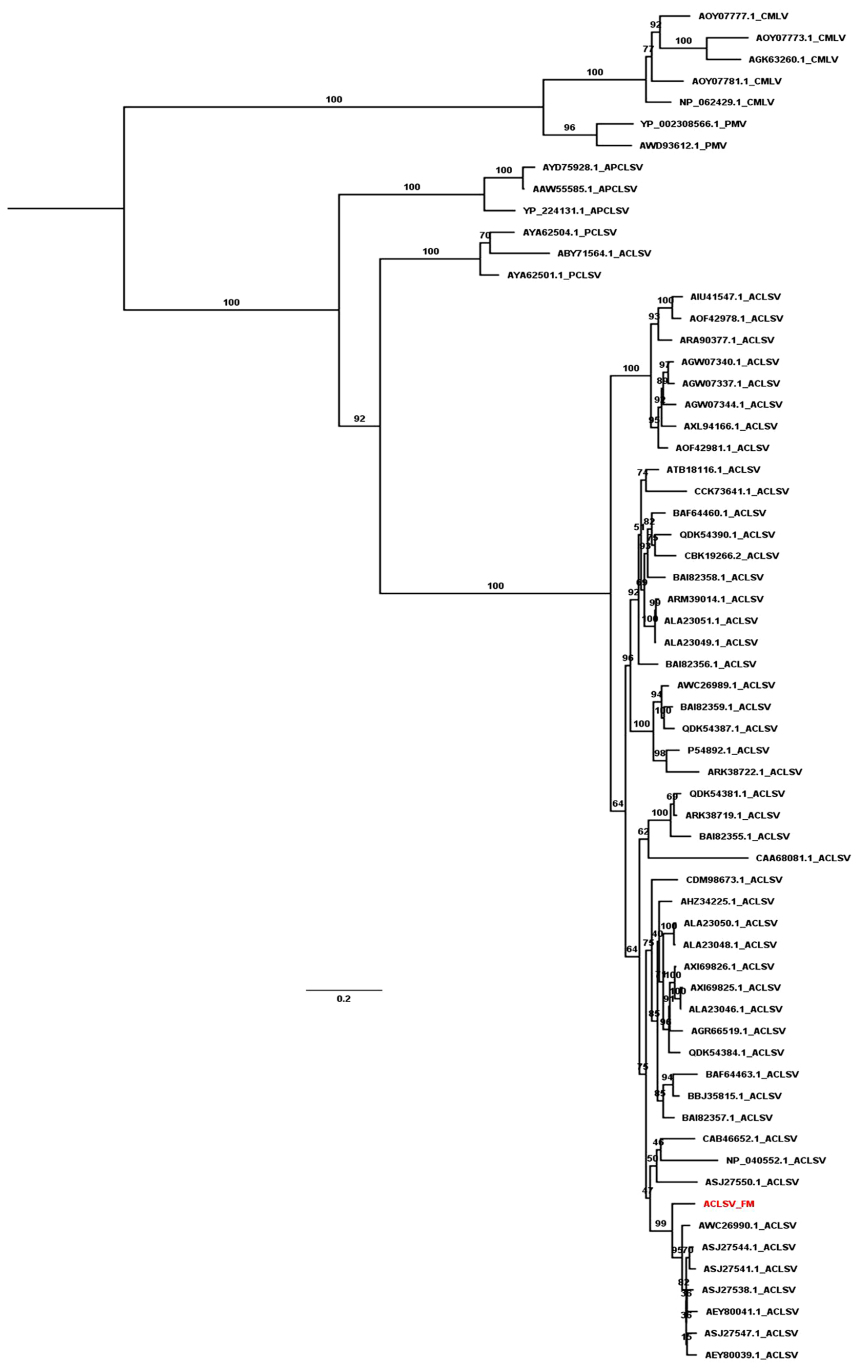
Maximum likelihood phylogenetic tree inferred from the MP sequences of known ACLSV isolates. MP sequences of cherry mottle leaf virus (CMLV), peach mosaic virus (PMV), and PCLSV were also used for phylogenetic tree construction. The phylogenetic tree was constructed using the IQ-TREE program with the FLU+I+G4 model. Ultrafast bootstrap with 1,000 iterations was indicated. The scale bar represents 0.2 substitutions/amino acid position.

We revealed the phylogenetic relationships of ACLSV and PNRSV by generating phylogenetic trees using the available sequences of CP and MP. Based on the CP protein sequences, the ACLSV isolate FM was closely related to ACLSV isolate SQ6 (AEZ03868.1) and SQ3 (AEZ03866.1) from peach in China (Fig. S1). The phylogenetic tree inferred from MP showed that most ACLSV isolates were in the same clade except the Ta Tao 5 isolate from the USA (ABY71564.1), which was grouped with two peach chlorotic leaf spot virus (PCLSV) isolates (Fig. 4). This result suggests that the Ta Tao 5 isolate could be a member of PCLSV rather than ACLSV in the same clade. The ACLSV isolate FM in this study was closely related to isolate PR1 (AWC26990.1) from *P. salicina* in Brazil, four isolates from peach in Korea, and two isolates from peach in China.

The phylogenetic tree generated using CPs of known PNRSV isolates showed that most PNRSV isolates were closely related to three possible clades (Fig. S2). The PNRSV isolate HW in this study was closely related to the isolate UN (AAF89715.1) from sour cherry in the Czech Republic. The phylogenetic tree generated using MPs of known PNRSV isolates displayed two different clades (Fig. S3). The PNRSV isolate HW was closely related to the isolate M7960 AlmIt.pre1 (CAC37342.1) from almond in Italy.

## Discussion

Many viruses and viroids infecting stone fruits—including peach, plum, apricot, and cherry—are being identified (Jo et al., 2018b; Kinoti et al., 2020; Komorowska, Hasiów-Jaroszewska & Czajka, 2020). Of the stone fruit trees, plum trees could be the second-most important trees for providing fresh fruits during the summer season in Korea. Despite the importance of plums, the viruses and viroids infecting plum trees have not been well-studied in Korea. In this study, we have identified viruses and viroids infecting six different plum cultivars derived from three different *Prunus* species by RNA-seq.

Initially, RNA-seq revealed six viruses and a viroid from plum transcriptomes. PCR-based approaches are frequently used to support NGS results; similarly, we conducted RT-PCR for the identified viruses using virus- and viroid specific primers. In many cases, the RT-PCR results were consistent with those of RNA-seq. However, RT-PCR confirmed infection of three viruses, namely ACLSV, PNRSV, and PBNSPaV, and one viroid (HSVd) in plum trees. There are several possible reasons for the discrepancy between the RNA-seq and RT-PCR. The first is primer sequences; due to the low sequence coverage of the identified viruses, we used known primer sequences instead of newly designed primers. If the genome of an infected virus shows strong sequence diversity, the binding affinity of the known primers can be very low. For example, the strong sequence diversity among Asian prunus viruses as well as ACLSV and APCLSV has been previously reported (Liberti et al., 2005; Marais, Faure & Candresse, 2016). The second possible reason is the low abundance of identified viruses in the plum sample. The third is contamination during NGS. During library preparation, each library is tagged with an index sequence. Multiple libraries are pooled into a single lane and sequenced by a single NGS platform. Index misassignments are frequently reported by some commercial NGS platforms (Li et al., 2019). In fact, our six plum libraries were sequenced together with six peach libraries (Jo et al., 2019, 2018b). Therefore, it is likely that the sequence reads associated with three of the viruses—LChV-1, PeVD, and PLPaV—were derived from the peach libraries (Jo et al., 2019, 2018b). We also identified several reads associated with PLPaV in other peach libraries. However, we confirmed that only one peach cultivar (Baekcheon) out of 10 peach cultivars was infected by PLPaV by RT-PCR (Jo et al., 2019). The virus identification with a few reads could be due to index misassignment from other libraries. We have carefully assumed that the LChV-1, PeVD, and PLPaV infections in the plum trees that were unsupported by the RT-PCR results could have been contaminants. Index misassignment (index hopping or swapping) using Illumina HiSeq system has been described in many previous studies (Costello et al., 2018; Griffiths et al., 2018; van Boheemen et al., 2020). Although the proportion of mis-assigned reads might be low, it is desirable for virus identification and diagnostics to confirm NGS results by other methods.

The most frequently identified viral pathogen infecting plum trees in Korea was HSVd, as confirmed by both RNA-seq and RT-PCR (Jo et al., 2017b). Of the viruses infecting plum trees, PBNSPaV was only identified in the EP plum cultivar (*P. domestica*), not the other *P. salicina* cultivars or *P. cerasifera*. By contrast, infection of PBNSPaV in *P. cerasifera* and *P. serrulata* has been previously reported in China (Cui et al., 2011) and Japan (Candresse et al., 2017). Moreover, a previous study identified two PBNSPaV isolates from *P. salicina* in China by the FLX454 system (Marais et al., 2014). Therefore, it seems that PBNSPaV infects a wide range of *Prunus* species. To date, the most frequently studied virus infecting plum is PPV. In particular, infection of PPV in *P. domestica* has been intensively reported in diverse countries (García et al., 2014). Recently, PPV has been reported in different stone fruits in Korea (Oh et al., 2017). Fortunately, we did not identify PPV in our examined plum trees.

One main advantage of NGS in virus identification might be that we can obtain complete or nearly complete genomes of identified viruses and viroids. However, we did not obtain any nearly complete genomes of identified viruses except HSVd in this study. Based on our experience, the likelihood of obtaining complete viral genomes by NGS might be highly correlated with the plant samples and preparation approaches (Pecman et al., 2017). When a plant is severely co-infected by many viruses, the viral replication within the plant might be high, resulting in the production of a high number of viral copies (Jo et al., 2017a). In addition, the nucleic acid isolation, sequencing platform, depth of sequencing, assembler software, and the skill of the researcher conducting all required experimental procedures are important factors for virus identification using NGS (Massart et al., 2017; Pecman et al., 2017).

Technically, it is impossible to enrich only viral nucleic acids from infected plant samples, although several research groups have suggested that the extraction of dsRNAs and small RNAs could enrich viral RNAs. Here, we used an mRNA library for RNA-seq instead of a library from dsRNAs or small RNAs. Our previous studies have demonstrated the usefulness of mRNA libraries for the identification of viruses and viroids with and without poly(A) tails (Jo et al., 2018a, 2017a, 2018b; Yoon et al., 2018). Compared to our previous study identifying viruses infecting peach trees (Jo et al., 2018b), the plum transcriptomes possessed few virus-associated reads, although we harvested plum leaf samples and peach leaf samples at the same time. Thus, in our study, sample preparation rather than sampling time might be a major factor causing failure to obtain as many virus-associated reads as possible. By contrast, several previous studies demonstrated the importance of sampling time for virus identification (Maliogka et al., 2018; Zotto et al., 1999). The fact that we obtained a smaller number of virus-associated reads from plum trees than from peach trees could be associated with the plant genome size or ploidy level. However, we obtained more virus-associated reads from European plum (*P. domestica*) with a hexaploid genome (2*n* = 6× = 48) than from *P. salicina* with a diploid genome (2*n* = 16) (Boonprakob et al., 2001; Zhebentyayeva et al., 2019). Based on our results, the ploidy level might not be related to the proportion of viral reads in plant transcriptomes. As suggested previously (Jo et al., 2015; Yoon et al., 2018), it seems that the proper selection of plant tissues and developmental stages could be important factors in increasing the proportion of virus-associated reads in the plant transcriptome.

## Conclusions

Taken together, we identified three viruses ACLSV, PBNSPaV, and PNRSV and one viroid (HSVd) infecting plum trees by RNA-seq and RT-PCR. In addition, we confirmed that the three viruses identified by RNA-seq could have been contaminants caused by index misassignment. Therefore, we suggest that careful confirmation by other methods be carried out in NGS-based virus identification.

## Supplemental Information

10.7717/peerj.9588/supp-1 Supplemental Information 1 Summary of virus-associated contigs and reads in each plum sample. The accession number of individual RNA-seq and the number of total reads and clean reads (trimmed reads). Clean reads were analyzed by PRINSEQ program.

10.7717/peerj.9588/supp-2 Supplemental Information 2 Summary of virus-associated contigs and reads in each plum sample. Virus-associated contigs were assembled by Trinity (v.2.8.6) assembler and viral reads were mapped on the reference genome using BBMap to calculate mapped bases, coverage, number of reads, and RPKM.
The names of plum samples were abbreviated as follows: Akihime (*Prunus salicina*) (AH), Gadam (*Prunus salicina*) (PG), Yeonsik (*Prunus salicina*) (PY), Hollywood (*Prunus cerasifera*) (HW), an unknown European plum (*Prunus domestica*) (EP), and Formosa (*Prunus salicina*) (FM).

10.7717/peerj.9588/supp-3 Supplemental Information 3 Maximum likelihood phylogenetic tree inferred from the CP sequences of known ACLSV isolates. The ACLSV isolate FM is indicated by red color. The CP of CMLV was used as an out-group. Ultrafast bootstrap with 1,000 iterations was indicated. The scale bar represents 0.5 substitutions/amino acid position.

10.7717/peerj.9588/supp-4 Supplemental Information 4 The maximum likelihood phylogenetic tree inferred from the CP sequences of known PNRSV isolates. The PNRSV isolate HW is indicated by red color. The CP of apple mosaic virus (ApMV) was used as outgroup. Ultrafast bootstrap with 1,000 iterations was indicated. The scale bar represents 0.5 substitutions/amino acid position.

10.7717/peerj.9588/supp-5 Supplemental Information 5 Maximum likelihood phylogenetic tree inferred from the MP sequences of known PNRSV isolates. The PNRSV isolate HW is indicated by red color. The MPs of lilac leaf chlorosis virus (LLCV), ApMV, and blueberry shock virus (BIShV) were used as outgroups. Ultrafast bootstrap with 1,000 iterations was indicated. The scale bar represents 0.5 substitutions/amino acid position.

## Additional Information and Declarations

### Data Availability

The following information was supplied regarding data availability: All raw data is available in the SRA database in NCBI: PRJNA295439.
