# Supplementary figures and images for: Identification of viruses infecting six plum cultivars in Korea by RNA-sequencing
Source: PeerJ. 2020 Jul 29;8:e9588. doi: 10.7717/peerj.9588 (PMC7395596; doi:10.7717/peerj.9588)

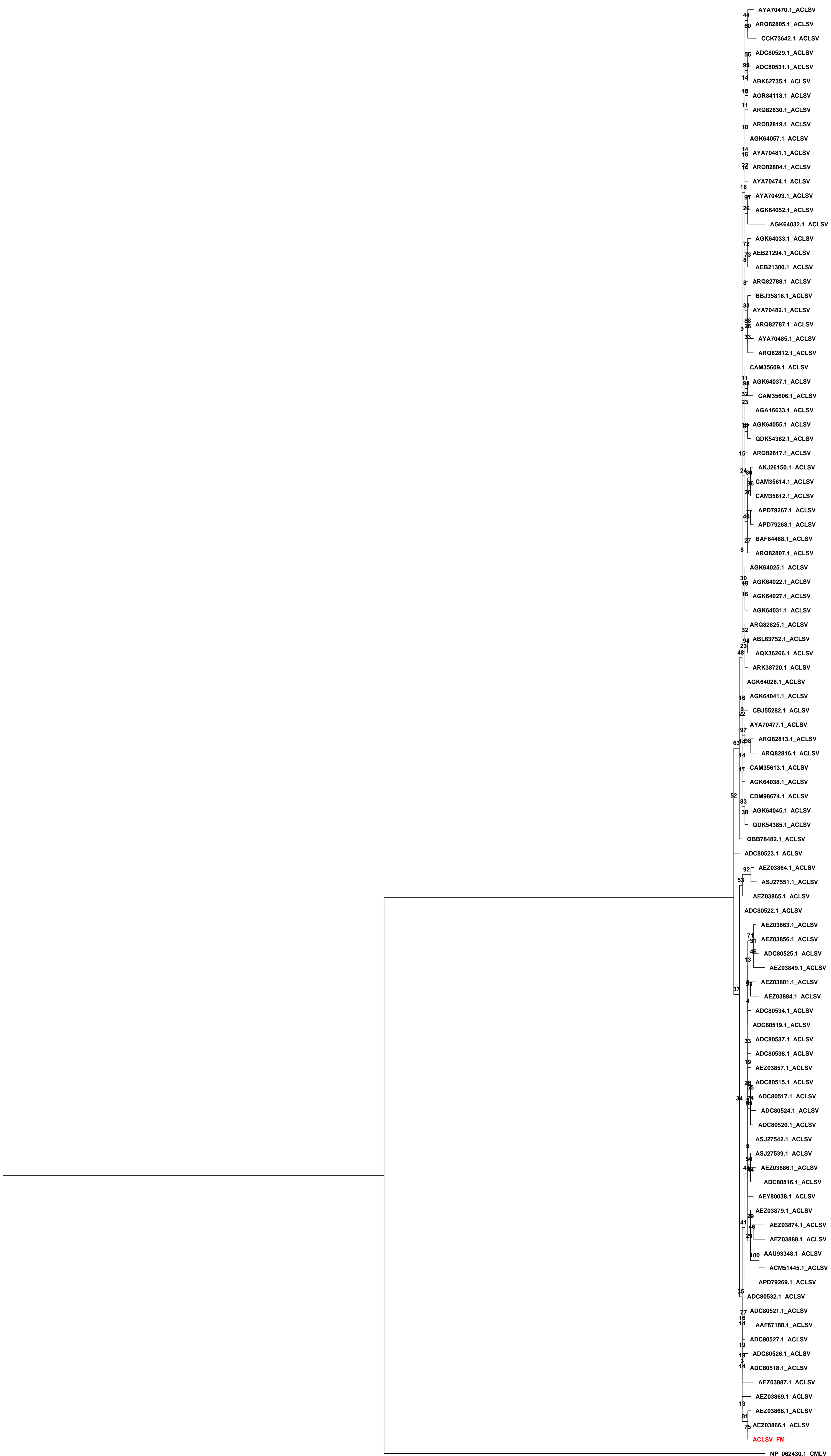

Supplement: Supplemental Information 3 — The ACLSV isolate FM is indicated by red color. The CP of CMLV was used as an out-group. Ultrafast bootstrap with 1,000 iterations was indicated. The scale bar represents 0.5 substitutions/amino acid position. [file peerj-08-9588-s003.pdf]

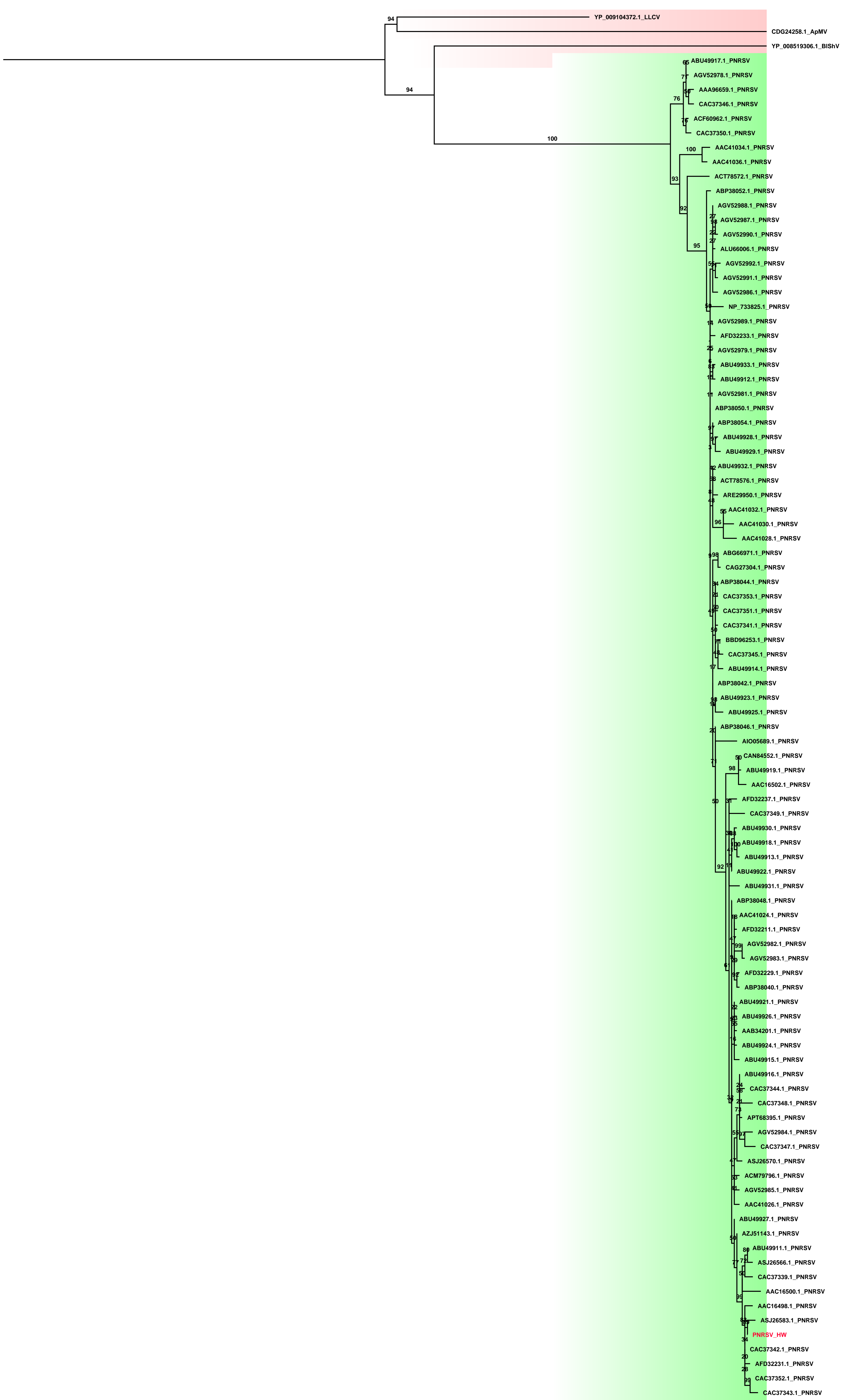

Supplement: Supplemental Information 5 — The PNRSV isolate HW is indicated by red color. The MPs of lilac leaf chlorosis virus (LLCV), ApMV, and blueberry shock virus (BIShV) were used as outgroups. Ultrafast bootstrap with 1,000 iterations was indicated. The scale bar represents 0.5 substitutions/amino acid position. [file peerj-08-9588-s005.pdf]
