# Supplementary material for: Identification of viruses infecting six plum cultivars in Korea by RNA-sequencing
Source: PeerJ. 2020 Jul 29;8:e9588. doi: 10.7717/peerj.9588 (PMC7395596; doi:10.7717/peerj.9588)
Supplement: Supplemental Information 4 — The PNRSV isolate HW is indicated by red color. The CP of apple mosaic virus (ApMV) was used as outgroup. Ultrafast bootstrap with 1,000 iterations was indicated. The scale bar represents 0.5 substitutions/amino acid position. [file peerj-08-9588-s004.pdf]

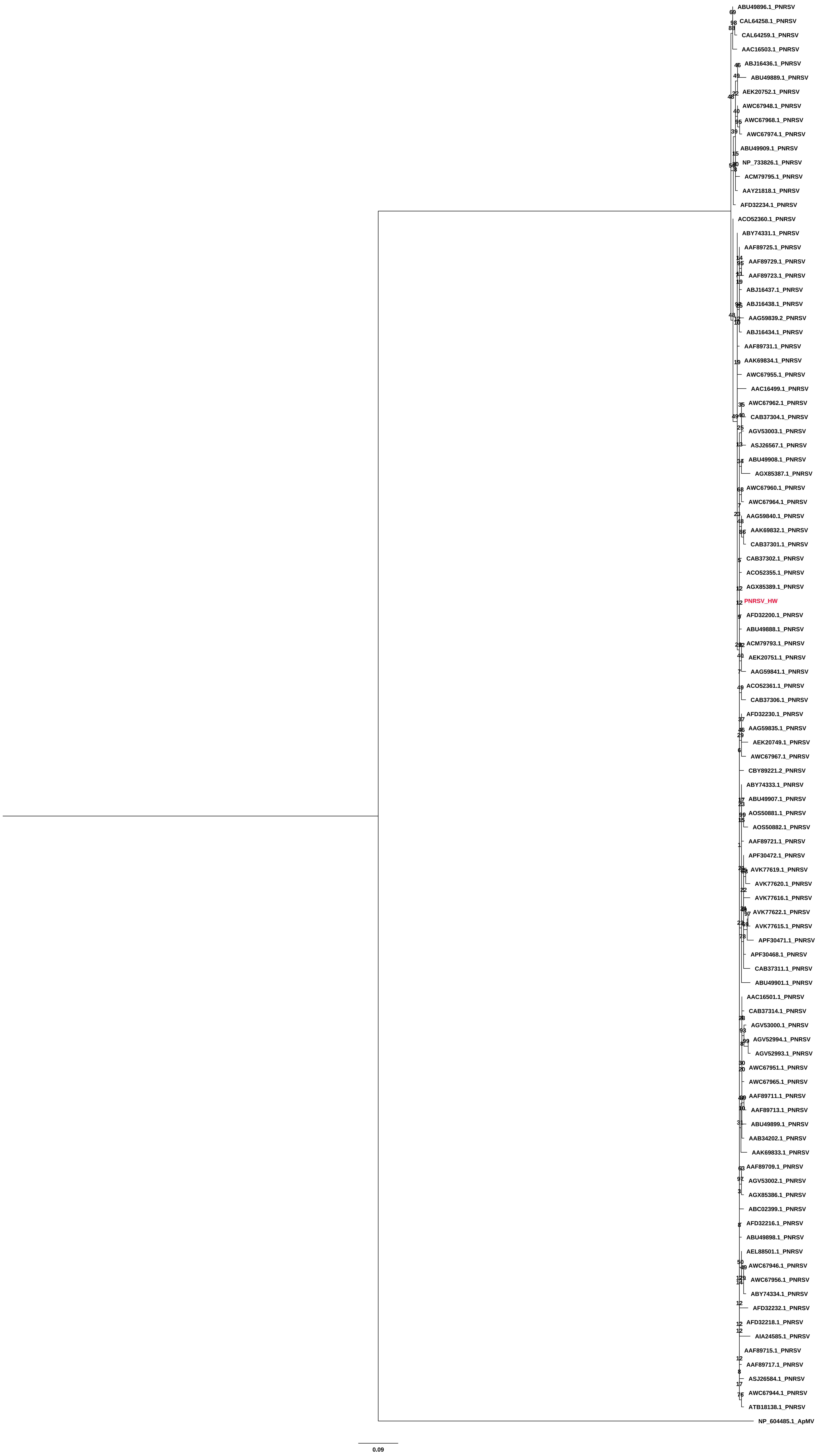
ABU49896.1_PNRSV
CAL64258.1_PNRSV
CAL64259.1_PNRSV
AAC16503.1_PNRSV
ABJ16436.1_PNRSV
ABU49889.1_PNRSV
AEK20752.1_PNRSV
AWC67948.1_PNRSV
AWC67968.1_PNRSV
AWC67974.1_PNRSV
ABU49909.1_PNRSV
NP_733826.1_PNRSV
ACM79795.1_PNRSV
AAY21818.1_PNRSV
AFD32234.1_PNRSV
ACO52360.1_PNRSV
ABY74331.1_PNRSV
AAF89725.1_PNRSV
AAF89729.1_PNRSV
AAF89723.1_PNRSV
ABJ16437.1_PNRSV
ABJ16438.1_PNRSV
AAG59839.2_PNRSV
ABJ16434.1_PNRSV
AAF89731.1_PNRSV
AAK69834.1_PNRSV
AWC67955.1_PNRSV
AAC16499.1_PNRSV
AWC67962.1_PNRSV
CAB37304.1_PNRSV
AGV53003.1_PNRSV
ASJ26567.1_PNRSV
ABU49908.1_PNRSV
AGX85387.1_PNRSV
AWC67960.1_PNRSV
AWC67964.1_PNRSV
AAG59840.1_PNRSV
AAK69832.1_PNRSV
CAB37301.1_PNRSV
CAB37302.1_PNRSV
ACO52355.1_PNRSV
AGX85389.1_PNRSV
PNRSV_HW
AFD32200.1_PNRSV
ABU49888.1_PNRSV
ACM79793.1_PNRSV
AEK20751.1_PNRSV
AAG59841.1_PNRSV
ACO52361.1_PNRSV
CAB37306.1_PNRSV
AFD32230.1_PNRSV
AAG59835.1_PNRSV
AEK20749.1_PNRSV
AWC67967.1_PNRSV
CBY89221.2_PNRSV
ABY74333.1_PNRSV
ABU49907.1_PNRSV
AOS50881.1_PNRSV
AOS50882.1_PNRSV
AAF89721.1_PNRSV
APF30472.1_PNRSV
AVK77619.1_PNRSV
AVK77620.1_PNRSV
AVK77616.1_PNRSV
AVK77622.1_PNRSV
AVK77615.1_PNRSV
APF30471.1_PNRSV
APF30468.1_PNRSV
CAB37311.1_PNRSV
ABU49901.1_PNRSV
AAC16501.1_PNRSV
CAB37314.1_PNRSV
AGV53000.1_PNRSV
AGV52994.1_PNRSV
AGV52993.1_PNRSV
AWC67951.1_PNRSV
AWC67965.1_PNRSV
AAF89711.1_PNRSV
AAF89713.1_PNRSV
ABU49899.1_PNRSV
AAB34202.1_PNRSV
AAK69833.1_PNRSV
AAF89709.1_PNRSV
AGV53002.1_PNRSV
AGX85386.1_PNRSV
ABC02399.1_PNRSV
AFD32216.1_PNRSV
ABU49898.1_PNRSV
AEL88501.1_PNRSV
AWC67946.1_PNRSV
AWC67956.1_PNRSV
ABY74334.1_PNRSV
AFD32232.1_PNRSV
AFD32218.1_PNRSV
AIA24585.1_PNRSV
AAF89715.1_PNRSV
AAF89717.1_PNRSV
ASJ26584.1_PNRSV
AWC67944.1_PNRSV
ATB18138.1_PNRSV
NP_604485.1_ApMV
0.09
